# Citizen science via social media revealed conditions of symbiosis between a marine gastropod and an epibiotic alga

**DOI:** 10.1038/s41598-020-74946-5

**Published:** 2020-11-12

**Authors:** Osamu Kagawa, Shota Uchida, Daishi Yamazaki, Yumiko Osawa, Shun Ito, Satoshi Chiba, The green-costumed snail’s citizen researchers, Emiko Kagawa, Akihiro Tamada, So Ishida, Junko Yoshida, Kazuki Kimura, Akiko Iijima, Takayuki Suenaga, Teruaki Momoi, You Kato, Satoshi Nikaido, Taeko Kimura, Shingo Kobayashi, Kazuo Niwa, Hirotaka Nishi, Haruto Fujita, Hideaki Kakihara, Shinichi Makino, Hiroe Suzuki, Akane Namikawa, Ryusei Yamakami, Kanae Higashi, Kota Watanabe, Taro Yoshimura, Isotomo, Mitsunori Sagara, Yuta Aoki, Ryoya Sugimoto

**Affiliations:** 1 Graduate School of Life Sciences, Tohoku University, Miyagi, Japan; 2 Wildlife Management Office Inc., Tokyo, Japan; 3 Center for Northeast Asian Studies, Tohoku University, Miyagi, Japan; 4 Amakusa Marine Biological Laboratory, Kyushu University, Kumamoto, Japan; 5 Guest House, Toy Bricks and Books in Manazuru, Kanagawa, Japan; 6 Osaka Museum of Natural History, Osaka, Japan; 7 Independent Researcher, Kumamoto, Japan; 8 Department of Biology, Kyungpook National University, Daegu, South Korea; 9 Department of Spanish and Portuguese, Kanda University of International Studies, Chiba, Japan; 10 Independent Researcher, Chiba, Japan; 11 Independent Researcher, Mie, Japan; 12 Independent Researcher, Tokyo, Japan; 13 Department of Life Sciences, Graduate School of Bioresources, Mie University, Mie, Japan; 14 Ehime Prefectural Science Museum, Ehime, Japan; 15 Hori Milk, Ishikawa, Japan; 16 Toyohashi Museum of Natural History, Aichi, Japan; 17 Nagasaki-Hokuyodai High School, Nagasaki, Japan; 18 Chiba City Buried Cultural Property Center, Chiba, Japan; 19 Independent Researcher, Aichi, Japan; 20 Laboratory of Marine Biology, Graduate School of Fisheries Sciences, Hokkaido University, Hokkaido, Japan; 21 Independent Researcher, Hyogo, Japan; 22 Independent Researcher, Fukui, Japan; 23 Faculty of Economics, Keio University, Tokyo, Japan; 24 Himeji City Science Museum, Hyogo, Japan; 25 Independent Researcher, Ehime, Japan

**Keywords:** Biodiversity, Community ecology, Ecological modelling, Evolutionary ecology, Wetlands ecology, Ecology

## Abstract

Environmental factors promote symbiosis, but its mechanism is not yet well understood. The alga *Pseudocladophora conchopheria* grows only on the shell of an intertidal gastropod *Lunella correensis*, and these species have a close symbiotic relationship which the alga reduces heat stress of the gastropod. In collaboration with general public, we investigated how environmental conditions alter the symbiotic interaction between the alga and the gastropod. Information about the habitats of each gastropod and images of shells was obtained from the Japanese and Korean coasts via social media. We constructed the hierarchical Bayesian model using the data. The results indicated that the proportion of shell area covered by *P. conchopheria* increased as the substrate size utilized by the gastropod increased. Meanwhile, temperature did not affect the proportion of *P. conchopheria* on the shell. These suggested that the alga provides no benefits for the gastropod on small substrates because gastropod can reduce the heat stress by diving into the small sediment. Further, the gastropod’s cost incurred by growing the alga on the shell seems to be low as the algae can grow even in cooler places where no benefits of heat resistance for gastropods. Different environments can yield variable conditions in symbiosis.

## Introduction

Symbiotic interactions provide a model to investigate how communities are developed^1–3^. Symbiosis includes mutualism, which benefits both organisms; commensalism, where one organism benefits and the other has no impacts; parasitism, where one organism is disadvantaged and one organism benefits; and amensalism, where one organism is harmed and the other receives no benefit^4^. However, the types of these symbiotic interactions vary over both space and time, depending on the environmental setting in which a host and symbiont interact^5,6^. These variations in symbiosis arise from different balances between the costs and benefits of the interactions in each environment^7^. Therefore, it is important to investigate how environmental factors promote specific symbiosis in understanding the evolutionary processes of symbiosis. In particular, a suitable model could be derived from conditional symbiosis systems in which symbiotic interactions change according to the environmental conditions^8^.

Symbiosis between epibionts (organisms that live attached to the surface of a host or substratum organism) and hosts is ubiquitous in marine ecosystems^1,9^. Several examples of positive or negative interactions have been found between epibionts and hosts^2,10^. We focused on an intertidal gastropod, *Lunella correensis,* and a shell-attaching alga, *Pseudocladophora conchopheria. P. conchopheria* has a unique symbiotic relationship in that it only grows on the shell of *L. correensis*^11–13^. Furthermore, the coverage of *P. conchopheria* varies according to the environment of the host gastropods^14^. Thus, the symbiotic relationship between these species is a conditional symbiosis. The interaction between these species has been reported to involve the water retention of *P. conchopheria* reducing the heat stress of *L. correensis* experienced during low tide. This suggests that the heat-resistance benefits from *P. conchopheria* vary among habitats^15^. However, the environmental factors contributing to the adhesion of *P. conchopheria* have only been examined locally (for example, in one bay^14^), and opinions on the environmental factors of the alga attaching to the shell of this gastropod differ between some studies^14,15^. No studies have examined the environmental conditions which symbiosis between *L. correensis* and *P. conchopheria* occurs by conducting extensive surveys covering the distribution area.

To address these issues, we focused on the “Citizen science” which is spreading in recent years in many fields. Citizen science can be a powerful tool for investigating the environmental effects on ecological traits through public participation and collaboration in scientific research. In the last decade, citizen science has contributed to the collection of large amounts of information and datasets^16,17^. In the field of ecology, citizen science is beginning to be recognized as a useful tool, as ecological studies often require the acquisition of a wide range of data or time series data^18^. Previous studies using citizen science have strongly focused on ecological information for conservation, such as the distribution of invasive species or the distribution of endangered species^19–23^. Some studies have also aimed to solve traditional problems in ecology by obtaining ecological data with the help of citizen science^16,24^. Developments in social media or social networking services have facilitated such efforts^25,26^. Additionally, citizen science not only enables efficient research, but also provides a means of dissemination of ecology and educational roles^27,28^.

**Figure 1:**
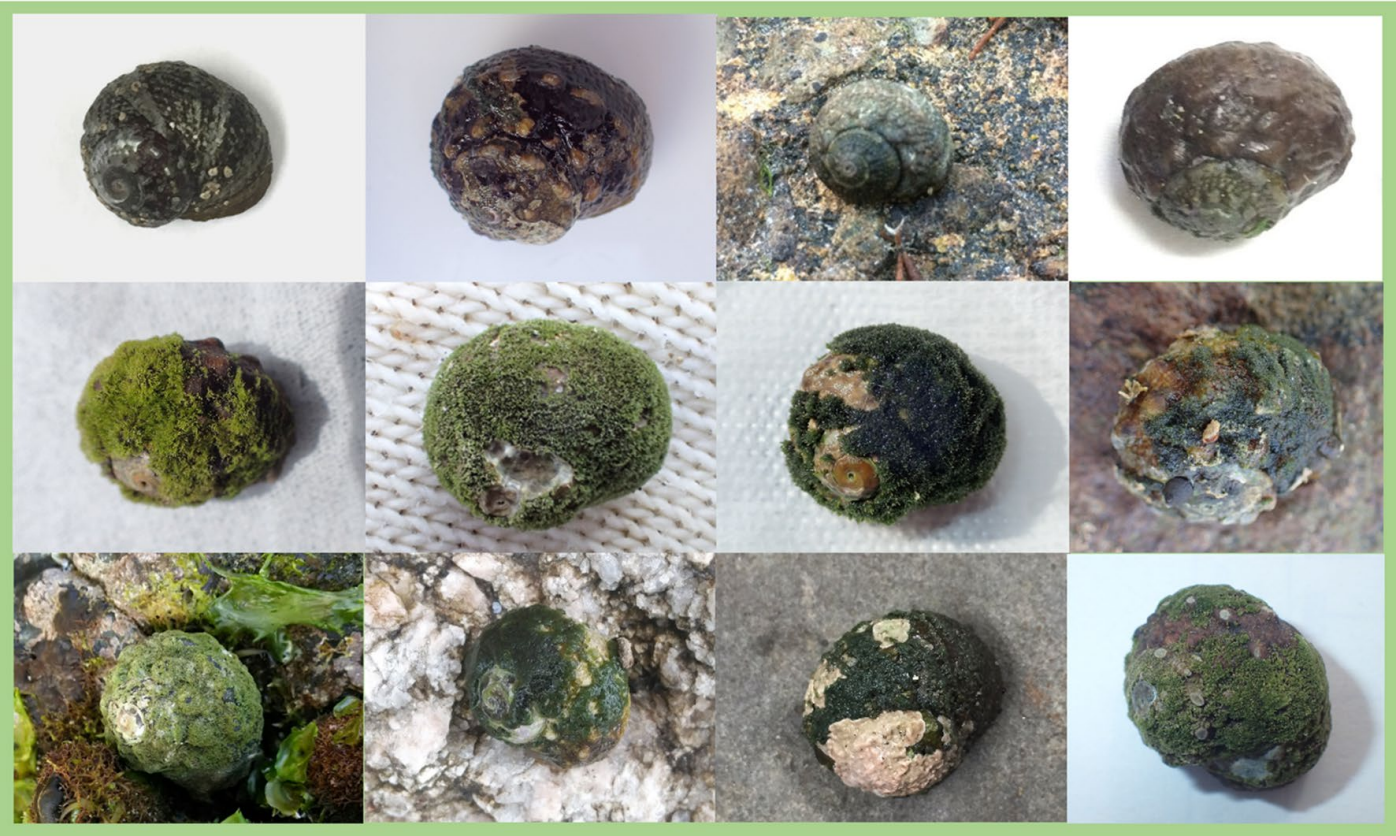
Images of *Lunella correensis* provided by citizens. The upper row is *L. correensis* without *Pseudocladophora conchopheria* on the shell. The middle row and lower row are *L. correensis* with shells that are entirely or partially covered by *P. conchopheria*.

The ecological traits of many organisms are maintained in biotic or abiotic environments^29^. Thus, ecological information is more useful when inter- or intraspecific interactions are taken into account. However, few projects have focused on such interactions between organisms in previous research using citizen science. Symbiosis is one of the common biological phenomena known to citizens (e.g., the relationship between clownfish and sea anemones is particularly well known). *L. correensis* is widely distributed along the coast of Japan and South Korea^12,30^ and is used by people as a fishery resource in some local areas^31^. Thus, the gastropod can easily be observed in the intertidal zone. In addition, *P. conchopheria* has a visually distinctive appearance because it covers the shells of *L. correensis* in a mat-forming manner (Fig. 1)^12^. Therefore, focusing on the symbiotic relationship between *L. correensis* and *P. conchopheria* is suitable for studying the biotic or abiotic interactions through a citizen science project.

The purpose of our study is to investigate the environmental conditions in which the symbiotic relationship between *L. correensis* and *P. conchopheria* occurs. In this study, we started a citizen science project (“The survey of *Lunella* gastropods and shell-adhering algae” in English; https://sites.google.com/view/sugai/) and collected images and environmental information related to the habitats of *L. correensis* via social media (Twitter, Facebook, Gmail, Google Forms). Based on the collected images of shell surfaces, the coverage of *P. conchopheria* was quantified. A hierarchical Bayesian model was constructed, and the environmental parameters that influence the coverage of *P. conchopheria* were obtained by a Markov chain Monte Carlo (MCMC) simulation.

## Results

**Figure 2:**
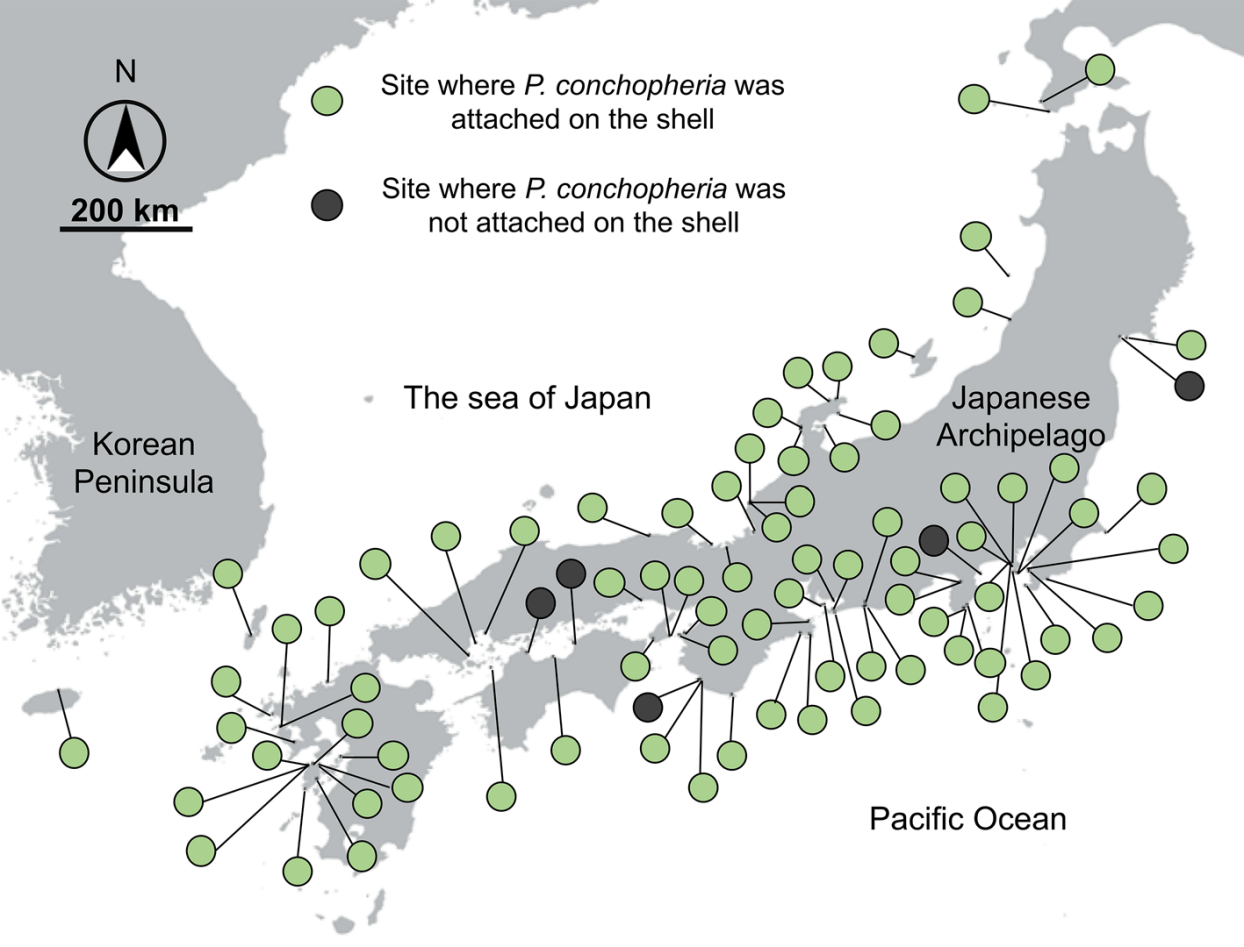
A map of 83 sites where *Lunella correensis* was discovered by the citizen science project. Green circles indicate the sites where *Pseudocladophora conchopheria* was attached on the shell of *L. correensis,* and black circles indicate where it was not attached to the shells. The map was created using Qgis 3.6.0-Noosa (https://qgis.org/ja/site/) and Map data is Natural Earth Free Vector and Raster Map Data.

**Figure 3:**
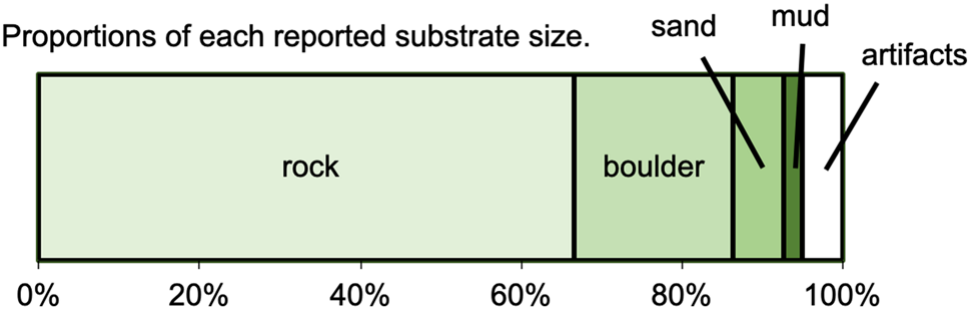
Proportions of each reported substrate size. For simplicity, points containing multiple substrate types were excluded from this graph. The results are from a total of 81 sites.

In total, 89 reports were collected through the citizen science project from April 2018 to Augast 2020 (Table S1). Most of these were reports of living *Lunella correensis* specimens, but three of them were for hermit crabs using shells from dead *L. correensis* gastropods (Table S1). *L. correensis* specimens with shells to which *Pseudocladophora conchopheria* adhered were confirmed in most sites (Figs. 1, 2, Table S1). However, there are a few sites where *P. conchopheria* was not found, and the proportions of *P. conchopheria* of attached to shells were various between reported sites (Fig. 1, Table S1). Rock was the most commonly reported type of substrate in the gastropods’ environments, while mud and sand were fairly rare in the reports (rock: 66.6%; boulder: 19.7%; sand: 6.2%; mud: 2.5%; artifacts: 4.9%; Fig. 3).

To evaluate the environmental parameters that affect the proportions of area covered by *P. conchopheria*, we conducted measurements of *P. conchopheria* coverage and applied a hierarchical Bayesian model with a MCMC simulation. The model included the wave fetch (an index of wave strength), substrate size and temperatures at each site, and we calculated the parameters of each environmental factor in relation to the algae coverage. In this analysis, 222 individuals from 66 sites were used (Table S1). The individuals were excluded from the analysis if their images had insufficient resolution or if multiple substrate sizes were reported. The shells used by the hermit crabs were also removed from this analysis. A site in South Korea was excluded from the analysis because there was no temperature data.

**Figure 4:**
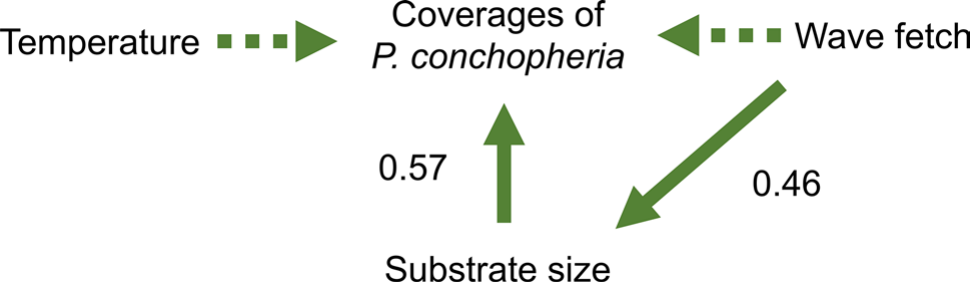
The result of the hierarchical Bayesian modelling. Dotted arrows indicate no effects. Solid arrows indicate effects, and the numbers indicate mean of each regression coefficient. The wave fetch did not have a direct effect on the coverage of *Pseudocladophora conchopheria*, but it did have an indirect effect on the coverage through the substrate size.

**Figure 5:**
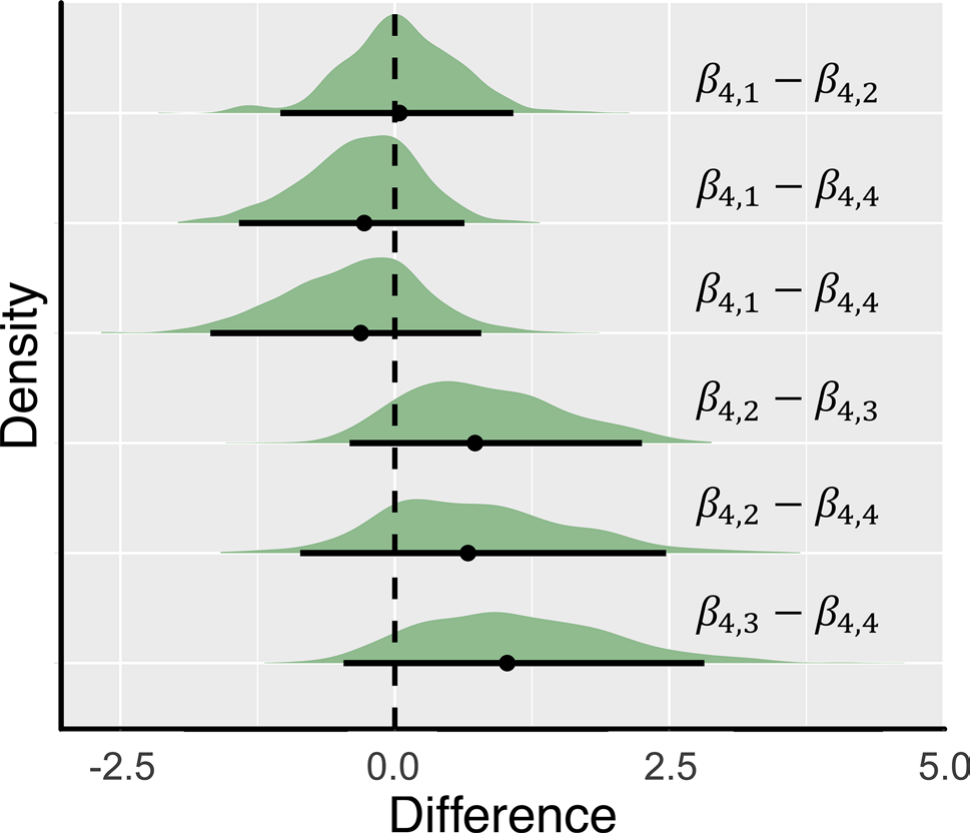
The results of the Bayesian t-test. The distribution of the differences for each parameter corresponds to the numerical expressions on the right. The dotted line is 0, the black points indicate the mean value of the difference, and the black lines indicate the 95% high density intervals.

As a result of the MCMC simulation, the posterior distribution for each parameter was obtained (Fig. 4, Table 1, Table S2). The regression coefficient of the substrate size ($\beta_{3}$) was 0.57 on mean, and values of 0 or less were not included in the 95% Bayesian confidence interval. As a result, the substrate size had a positive effect on the coverage of *P. conchopheria*. On the other hand, the regression coefficient of wave fetch ($\beta_{2}$) was − 0.08 on mean, and 0 was included in the 95% Bayesian confidence intervals. This result showed that the wave fetch had almost no effect on the coverage of *P. conchopheria*. The Bayesian model was incorporated to test whether the wave fetch affects substrate size. The wave fetch regression coefficient ($\alpha$) was 0.46 on mean, and values of 0 or more were not included in the 95% confidence interval. Thus, the wave fetch had a positive effect on the substrate size. Therefore, the wave fetch had no direct effect on the coverage of *P. conchopheria*, but it indirectly had a positive effect via substrate size (Fig. 4). The regression coefficient for temperature ($\beta_{4}$) was − 0.06 on mean, and 0 was included in the 95% confidence interval. As a result of a Bayesian t-test, the difference of the posterior distribution for temperature between different substrate sizes included in 0 in the 95% confidence interval (Fig. 5, Table S2). Thus, there was no effect of temperature on the coverage of *P. conchopheria*. In addition, the effect of temperature showed no difference between substrate sizes.

**Table 1:** Results of hierarchical Bayesian modeling using MCMC.

Parameter	Mean	S.D	95% Bayesian confidence interval	
			Lower (2.5%)	Upper (97.5%)
$\alpha$	0.46	0.15	0.19	0.77
$\beta_{1}$	− 1.45	1.06	− 3.50	0.61
$\beta_{2}$	− 0.08	0.19	− 0.45	0.31
$\beta_{3}$	0.57	0.29	0.01	1.13
$\beta_{4}$	− 0.06	0.83	− 1.73	1.73

## Discussion

In this citizen science project, citizens reported instances of *Lunella correensis* with *Pseudocladophora conchopheria* attached to it shell in most sites. The symbiotic relationship between *L. correensis* and *P. conchopheria* is likely to be ubiquitous in their distribution area. We detected environmental conditions under which symbiosis between the gastropod and algae was maintained. The proportions of *P. conchopheria* on shells became higher as the substrate size increased. *L. correensis* is commonly found on rocky shores^12,31,32^, and in the present study, it was more commonly found to inhabit rocky shores with larger substrates (i.e., rocks) than smaller substrates (i.e., sand and mud). Since the shells of living *L. correensis* are the only habitat for *P. conchopheria*^32^, this alga needs to attach efficiently to the shells of these gastropods in a wide range of various environments. *P. conchopheria* must be distributed where there are large populations of *L. correensis* in order to increase the probability of attaching to their shells. Thus, the congruence of environmental preference between *L. correensis* and *P. conchopheria* may be explained by the adaptation of *P. conchopheria*.

As an alternative hypothesis, the life history of the host gastropods may limit the growth of algae^33,34^. *L. correensis* often burrows into the sand or mud during low tide^15,35^. The gastropod’s behaviour of hiding under the substrate depends on the substrate size. Burrowing into the mud or sand may inhibit the growth of *P. conchopheria* through the inhibition of photosynthesis, through physical peeling, or through suppression of the establishment of zoospores of *P. conchopheria*. Therefore, it is possible that the effects of different substrate sizes on the behaviour of *L. correensis* affected the proportion of *P. conchopheria* on the shell. In the future, a more detailed mechanism of how the growth of *P. conchopheria* is restricted to the shell needs to be investigated.

The evolution of symbiosis may result from a balance among the benefits and costs between symbionts and hosts^5,6^. Many examples of interactions are well known between attached organisms and hosts^1,36^. However, many of these interactions are negative and are costly for the host^1,38–42^. Several hosts have evolved chemical, physical, or other bio-mediated defence systems to protect themselves from epibiotic attachment^1,43,44^. Therefore, many adherent organisms are generalists that do not have epibiotic relations or host specificity^45^. In order for adherent organisms to acquire host specificity, they may need to provide positive or neutral effects for the host^46^. A recent study showed that *P. conchopheria* provides benefits to the host *L. correensis* by reducing its heat stress during low tide^15^. On the other hand, *L. correensis* seems to avoid heat stress during low tide by burrowing into sand or mud^15,35^. The benefits from *P. conchopheria* may not be as great in habitats with small substrate size such as mud or sand^15^. The difference in benefit between substrate sizes may explain the difference in proportions of *P. conchopheria* on the shells.

Additionally, costly traits are likely not to be maintained unless supplemented by other benefits^47^. If the attachment of *P. conchopheria* is costly for the gastropod host, *P. conchopheria* will not be maintained on the shells in the cooler areas because it could provide less benefit for the gastropods than in hot areas. However, in this study, there was no effect of temperature on proportions of *P. conchopheria* on the shells when considering substrate size. Many epibiotic algae are costly for hosts. For example, they can increase the possibility of detachment of the host gastropod from the substrate rock due to wave resistance^1,38^ or increase vulnerability due to perforation by the algae at the root attachment points^48^. However, *P. conchopheria* is smaller than other algae in the family Cladophoraceae, which is closely related and uses abiotic substrates^11^, and the “roots” of *P. conchopheria* do not reach the inner layer of the shell of *L. correensis*^14^. A previous study also showed that the amount of *P. conchopheria* had no effect on the mortality of *L. correensis* when reared in a laboratory^32^. Therefore, *P. conchopheria* on the shell may be maintained in a commensalist relationship by reducing the cost for *L. correensis* in cooler areas where the benefit of heat resistance is not required.

Several studies showed that epibionts on gastropod shells defend the host from predators by using visual, physical, and chemical defence systems^1^. The presence of predators may have an important impact on building symbiotic relationships between epibionts and hosts^49,50^. *P. conchopheria* may defend *L. correensis* from its predators. There are a few predators of *L. correensis,* such as malacophagous crabs and gastropods (Fig. S1)^51,52^. An intertidal gastropod of Turbinidae are predated by starfish in Japan^53^, and therefore, *L. correensis* is potentially predated by starfish. The density of these malacophagous predators may differ among different environments^54^. Therefore, since the predator density is related to the substrate size, the proportions of *P. conchopheria* may differ with changes in substrate size. However, biotic factors such predation are effective in the lower part of the intertidal zone, whereas physical factors are effective in the upper part of the intertidal zone, which *L. correensis* and *P. conchopheria* inhabit^55^. Predation by birds is an important factor in the upper intertidal zone^56^, but, no bird feeding on *L. correensis* have been reported^57,58^. On the other hand, in terms of their role in predator defense, *P. conchopheria* may also benefit from *L. correensis*. In the intertidal zone, the presence of grazers has both direct and indirect effects on algae communities^59,60^. *P. conchopheria* is smaller in size than its related species^11^. In the laboratory, *P. conchopheria* is eaten by *L. correensis* and disappears from the shells of *P. conchopheria* when *P. conchopheria*-covered *L. correensis* is reared at a high density^13^. These suggest that *P. conchopheria* is eaten by grazers. *P. conchopheria* may be protected from grazers by being attached to the shell. However, it remains questionable whether predation actually has a strong effect and evolutionary influence on the proportions of *P. conchopheria*. In any case, the predator defense function by *P. conchopheria* and *L. correensis* has not been confirmed respectively, requires clarification in future research.

Several researchers have focused on the system of *L. correensis* and *P. conchopheria* to elucidate environmental factors for the adhesion of this alga^14,15,61^. One study showed that the proportion of *P. conchopheria* was higher on gastropods in an inner bay, suggesting that the substrate size and the wave strength influence the algal coverage^14^. Another study suggests that substrate size was important in the conditions of attachment of *P. conchopheria* rather than wave strength^15^. However, the survey sites of these studies were in small areas, and it is unclear what environmental factors affect the coverage of *P. conchopheria* because wave height is correlated with substrate size in these survey sites. Our results revealed that the proportions of *P. conchopheria* coverage are not affected by wave fetch, but they are affected by substrate size. Thus, these environmental factors were evaluated separately by conducting extensive surveys with support from the general public and by constructing the hierarchical Bayesian model. Without collaboration with the general public, it would be difficult to obtain such a wide range of data. Thus, this study demonstrates the importance of the contributions of citizen science on ecological or evolutionary studies.

## Methods

### Citizen science project

A citizen science project (“The survey of *Lunella* gastropods and shell-adhering algae” in English) was started in April 2018. The project needed recognition by many citizens, so we announced it on a website (https://sites.google.com/view/sugai/), Twitter (https://twitter.com/lunellacoreens), and Facebook (https://www.facebook.com/lunellacoreensis/). All information was collected using Google Forms (https://forms.gle/8WCVVmJMDMvjAEtaA), e-mail (lunellacoreensis@gmail.com) and Twitter. On Twitter in particular, the posted information was obtained by tweets with the hashtags “#Sugai Hakken!” in Japanese and “#discovered *L. correensis*!” in English. On these social media sites, we asked citizens to take photos of *Lunella correensis* in nature and to provide information about the locations in which they were discovered. In order to reduce the photography bias and to measure the coverage of *Pseudocladophora conchopheria*, we designated a method where the information providers were instructed take a photograph of gastropods from above the shell with the shell aperture facing down. Geotagged photos were also recommended. In addition, for identification of *L. correensis*, we asked the general public collaborators to check for the presence or absence of thick and round operculum. We showed on the website and SNS, as examples, photos of *L. correensis* shells with and without *P. conchopheria*. In particular, arrows and photos were used to illustrate the snail operculum on the website and SNS sites to draw attention to the snail's operculum. If the public collaborators could not identify the gastropods, we identified them from the posted images. The adhesion of *P. conchopheria* was checked using the posted image by the authors. Additionally, we asked about the day on which the specimen was discovered, the substrate size of the area, the location information, and the nickname or the name of the information provider. The location information was latitude and longitude or an address, and if the address was unclear, the authors re-contacted the provider and asked for the detailed location. The information on substrate type was provided by the provider in five categories [rock, boulder, sand, mud, and artificial substrates (tetrapods, etc.)]. We provided a sample photo of each environment on our website, Twitter, and Google Forms. We also received photos of the environment. Consent for use of the provided content in research data was obtained from all providers in all posts. All images and nicknames used in this paper have been approved by the providers.

### Measurement of proportions of *P. conchopheria* on the shells

The density of epibionts on the host alters the interactions that the host accepts from the epibiont^33^, so we calculated the proportions of *Pseudocladophora conchopheria* on the shells. To measure the coverage of *P. conchopheria*, the total area of the shell surface and the area covered by *P. conchopheria* were measured on the images provided. For the measurement, the backgrounds of images were deleted using the selection tools of GIMP 2, the image contrast was adjusted with the program’s colour tools, and then each area was measured using ImageJ^62^. The coverage was obtained by dividing the area covered by *P. conchopheria* by the total shell area. These methods were conducted with reference to Kagawa and Chiba 2019^15^.

### Acquisition and handling of data used for the hierarchical Bayesian modelling

We constructed hierarchical Bayesian models to determine how environmental parameters affect the coverage of *Pseudocladophora conchopheria*. All variables were obtained and processed for each model. Previous studies showed that the degree of coastal openness affects the coverage of *P. conchopheria*, suggesting that the wave strength is one of important factors^14^. Therefore, the wave fetch was calculated as an index of wave strength based on the latitude and longitude information collected by the citizen science project^63–66^. Lines with lengths of 100 km were placed in 32 directions around the survey points on a map. The distance at which each line contacted the opposite shore from the survey points was measured, and the log-converted values of the total of all distances were used as wave fetch of the survey point. The wave fetch of all sites was calculated using the package fetchR^67^ in R version 3.5.2^68^.

The sediment environment may also influence the coverage of *P. conchopheria*. *Lunella correensis* living in an environment of sand or mud sediment dives into the sediment^35^, which may indirectly or directly decrease the coverage of *P. conchopheria*^15^. Considering that *L. correensis* can dive into substrates of smaller size, we used ordinal variables for the substrate size (mud: 1, sand: 2, boulder: 3, rock: 4) in the modelling. The artificial substrate was excluded as a substrate size in the modelling, because it includes structures of various sizes. The site with multiple substrates reported were excluded from the analysis.

In addition, *P. conchopheria* has an effect of reducing the heat stress for *L. correensis* at low tide in summer^15^, which may affect the coverage as well. We obtained mean value for 30 years of maximum temperature in each day in August from the National Land Numerical Information’s download service^69^. This is a mesh data of 1 square kilometre that indicate temperature. By superimposing this mesh data on the map with the survey points, the values for the section to which the survey sites belonged were obtained using the point sampling tool of QGIS 3.6. The coverage of *P. conchopheria* may be affected by seasonal changes^14,32^, so the season in which *L. correensis* was discovered at each site was obtained from citizen science data. According to the Japan Meteorological Agency, the months of March, April, and May are spring, June, July, and August are summer, September, October, and November are autumn, and December, January, and February are winter^70^. Thus, the month in which a specimen was discovered was replaced with a categorical variable for each season (spring: 1, summer: 2, autumn: 3, winter: 4).

Poor quality photos, photos that were not taken at the recommended angles, and photos of hermit crabs were excluded from the analysis. Korean sites were excluded from the analysis because temperature data were not available in Korea.

### Model construction for the evaluation of environmental parameters affecting coverage of *P. conchopheria*

We constructed a hierarchical Bayesian model to evaluate environmental parameters affecting the coverage of *Pseudocladophora conchopheria*. The coverage of *P. conchopheria* was considered as a continuous variable ranging from 0 to 1, so we subjected it to beta regression^71^.

$$Coverage_{i} \sim Beta\left( {\phi \cdot \theta_{i} , \phi \left( {1 - \theta_{i} } \right)} \right)$$

$$logit\left( {\theta_{i} } \right) = \beta_{1} + \beta_{2} \cdot fet_{i} + \beta_{3} \cdot sub_{i} + \beta_{4, k} \cdot tem_{i} + \varepsilon_{site} + ssn_{t}$$

$$i = 1, 2 \ldots ,222$$

$$k = 1:{\text{mud}},2:{\text{sand}},3:{\text{boulder}},4:{\text{rock}}$$

$$site = 1, 2 \ldots ,66$$

$$t = {\text{spring}},{\text{summer}},{\text{autumn}},{\text{winter}}$$

In this model, $logit\left( {\theta_{i} } \right)$ is the link function of the beta distribution, exp(x)/(1 + exp(x)), and $\beta_{1}$ is the intercept of this model. $fet_{i}$ is the wave fetch value of the $i$ th gastropod, $\beta_{2}$ is the regression coefficient of $fet_{i}$, $sub_{i}$ is the substrate si of the $i$ th gastropod, $\beta_{3}$ is the regression coefficient of $sub_{i}$, $tem_{i}$ is the average annual maximum temperature of the $i$ th gastropod, and $\beta_{4, k}$ is the regression coefficient of $tem_{i}$. Furthermore, to consider the effect of site heterogeneity, the model included a random effect, $\varepsilon_{site}$, which has a normal distribution with a mean of 0 and variance $\sigma_{site}^{2}$. In consideration for seasonal changes, $ssn_{t}$ was included as a fixed effect.

$$\beta_{4, k} = \beta_{4} + \varepsilon_{k}$$

Differences in microhabitats lead to different thermal environments^35,72^. Assuming that the effect of temperature could vary according to the substrate size, we added a random effect of substrate size to $\beta_{4}$. The random effect, $\varepsilon_{k}$, has a normal distribution with a mean of 0 and variance $\sigma_{K}^{2}$.

$$sub_{i} \sim ordered\;logistic\left( {c, \alpha \cdot fet_{i} } \right)$$

The wave strength affects the substrate size^73^, so we hypothesized that the wave fetch could affect the coverage of *P. conchopheria* through the substrate size indirectly. Since the substrate size is considered as an ordinal variable, ordered logistic regression was performed. $\alpha$ is the coefficient value of $fet_{i}$, and $c$ is a threshold parameter (numbers of categories of the dependent variable minus 1).

### Parameter calculation

Parameters were calculated using Markov chain Monte Carlo (MCMC) simulations within a Bayesian framework. We assumed non-informative distributions to be the prior distributions of all parameters. $\beta_{1}$, $\beta_{2}$, $\beta_{3}$, $\beta_{4}$, $ssn_{t}$ $\alpha$, and c had normal distributions with a mean of 0 and variance of 100^2^. $\sigma_{site}$ and $\sigma_{K}$ had a uniform distribution with the interval [0, 15]. $\phi$ is a Cauchy distribution with 0 as the location parameter and 5 as the scale parameters. We conducted three parallel chains, each with 2000 MCMC simulations, an initial burn-in phase of 1000 iterations, and sampling rate of 5 times. All variables were standardized using Z-scores to eliminate numerical instabilities and improve the mixing of each chain^74^. All MCMC chains were confirmed to converge by low levels of auto-correlation and Gelman and Rubin diagnostics^75^.

Bayesian t-test was carried out to investigate whether the influence of temperature on the coverage of *Pseudocladophora conchopheria* differs between substrate sizes^76^. The posterior distribution of the differences between $\beta_{4, k}$ and the effect size were calculated. When the 95% Bayesian confident interval of the posterior distribution is sufficiently above 0, the effect of the temperature on the coverage of *P. conchopheria* differs between the substrate sizes. All of the above calculations were done using the package R-stan in R version 3.5.2^68^.

## Supplementary information

Supplementary Information
