# Supplementary material for: Citizen science via social media revealed conditions of symbiosis between a marine gastropod and an epibiotic alga
Source: Sci Rep. 2020 Nov 12;10:19647. doi: 10.1038/s41598-020-74946-5 (PMC7665050; doi:10.1038/s41598-020-74946-5)

Supplementary information

Citizen science via social media revealed conditions of symbiosis between a marine gastropod and an epibiotic alga

Osamu Kagawa[1*], Shota Uchida[1, 2], Daishi Yamazaki[3], Yumiko Osawa[4], Shun Ito[1] & Satoshi Chiba[1, 3] The green-costumed snails' citizen researchers[1]

[1]*Graduate school of Life Sciences, Tohoku University, Miyagi, Japan*

[2]*Wildlife Management Office Inc., Tokyo, Japan*

[3]*Center for Northeast Asian Studies, Tohoku University, Miyagi, Japan*

[4]*Amakusa Marine Biological Laboratory, Kyushu University, Kumamoto, Japan*

Correspondence and requests for materials should be addressed to O. Kagawa (email: ok.osamukagawa@gmail.com)

Supplementary Table S1

Information collected in this project.

Posted ID	Discovery date	Locality (city and prefecture)	Latitude	Longitude	Substrate	Adhesion of *P. conchopheria*	Number of snails with *P. conchopheria*	Number of snails without *P. conchopheria*	Use within HBM analysis	Note
1	2017/5/27	Futtsu, Chiba	35.186169	139.817564	Rock	adhesion	4	0	○	
2	2017/4/23	Kamogawa, Chiba	35.055822	140.073222	Rock	adhesion	4	0	○	
3	2018/1/14	Tokitsu, Nagasaki	32.842347	129.851229	Rock	adhesion	3	0	○	
4	2017/9/8	Hayama, Kanagawa	35.259763	139.575175	Rock	adhesion	1	2	○	
5	2018/4/9	Obama, Fukui	35.490691	135.720108	Rock	adhesion	13	0	○	
6	2017/5/3	Matsumae, Hokkaido	41.417693	140.088362	Rock	adhesion	1	0	×	
7	2018/4/15	Wakayama, Wakayama	34.307243	135.077897	Boulder	adhesion	1	1	○	
8	2018/4/14	Imabari, Ehime	34.062924	133.005869	Artifact	No adhesion	0	3	○	
9	2018/4/28	Niihama, Ehime	34.004032	133.369868	Rock	adhesion	3	0	○	
10	2018/4/22	Choshi, Chiba	35.692235	140.865612	Rock	adhesion	1	0	○	
11	2018/4/30	Misaki, Osaka	34.331410	135.153549	Rock	adhesion	1	0	○	
12	2018/4/30	Amakusa, Kumamoto	32.203799	129.995892	Rock	adhesion	3	0	○	
13	2018/4/16	Kosai, Shizuoka	34.685332	137.584742	Boulder	adhesion	1	0	○	
14	2018/4/30	Hamamatsu, Shizuoka	34.748967	137.597407	Boulder	adhesion	1	0	○	
15	2018/5/3	Shima, Mie	34.312935	136.848002	Boulder	adhesion	2	0	○	

16	2018/5/5	Hiroshima, Hiroshima	34.325662	132.441451	Rock	adhesion	1	0	○
17	2018/5/5	Shirahama, Wakayama	33.696800	135.362700	Rock, Boulder	adhesion	4	0	○
18	2018/5/20	Noto, Ishikawa	37.307293	137.236447	Rock	adhesion	18	0	○
19	2018/5/18	Amakusa, Kumamoto	32.551766	130.109722	Rock	adhesion	1	1	○
20	2018/5/14	Amakusa, Kumamoto	32.537497	130.192379	Sand	adhesion	2	0	○
21	2018/5/14	Amakusa, Kumamoto	32.540352	130.112099	Rock	adhesion	1	0	○
22	2018/5/14	Amakusa, Kumamoto	32.520931	130.031409	Rock, Boulder, Sand	adhesion	3	0	○
23	2018/5/14	Amakusa, Kumamoto	32.525063	130.038586	Boulder	adhesion	2	1	○
24	2018/5/14	Amakusa, Kumamoto	32.528075	130.034017	Boulder, Sand, mud, Artifact	adhesion	1	0	○
25	2018/5/17	Matsuyama, Ehime	33.852877	132.522118	Boulder	adhesion	1	0	○
26	2018/5/26	Iwakuni, Yamaguchi	34.034951	132.218138	Rock, Boulder, Sand	adhesion	1	0	○
27	2018/5/27	Matsunagi, Ishikawa	37.506599	137.204058	Rock	adhesion	3	0	○
28	2018/6/12	MinamiIse, Mie	34.328773	136.719189	Rock	adhesion	2	0	○

29	2018/5/19	Amakusa, Kumamoto	32.335662	130.160184	Artifact	adhesion	1	0	○
30	2018/5/19	Amakusa, Kumamoto	32.335662	130.160184	Artifact	adhesion	1	0	○
31	2018/5/20	Amakusa, Kumamoto	32.335662	130.160184	Rock	adhesion	3	0	○
32	2018/6/3	Tahara, Aichi	34.604813	137.189439	Artifact	adhesion	2	0	○
33	2018/6/16	Minamichita, Aichi	34.701679	137.004583	Rock	adhesion	1	0	○
34	2018/6/13	Manazuru, Kanagawa	35.140219	139.162566	Boulder	No adhesion	0	1	○
35	2018/4/20	Hayama, Kanagawa	35.269965	139.569806	Rock	adhesion	1	0	×
36	2018/7/8	Fukuoka, Fukuoka	33.659367	130.304596	Rock	adhesion	7	0	○
37	2018/7/16	Shirahama, Wakayama	33.693903	135.351521	Rock	adhesion	2	0	○
38	2018/7/18	Kami, Hyogo	35.661465	134.672752	Rock	adhesion	3	0	○
39	2018/7/19	Kami, Hyogo	35.661465	134.672752	Rock	adhesion	7	0	○
40	2018/8/26	Otake, Hiroshima	34.186581	132.315894	Rock	adhesion	1	0	○
41	2018/6/2	Toba, Mie	34.495401	136.837075	Boulder	adhesion	5	0	○
42	2018/7/16	Gamagoori, Aichi	34.772172	137.167482	Rock	adhesion	1	0	○
43	2018/8/4	Uki, Kumamoto	32.644602	130.482062	Boulder	adhesion	1	0	○
44	2018/8/28	Shimoda, Shizuoka	34.667590	138.941271	Boulder	adhesion	1	0	○
45	2018/8/29	Shimoda, Shizuoka	34.667360	138.939176	Rock	adhesion	3	0	○
46	2018/5/14	Matsushima, Miyagi	38.351789	141.058980	mud	No adhesion	0	2	○
47	2018/9/22	Sado, Niigata	38.074292	138.243979	Rock	adhesion	1	1	○
48	2018/8/26	Hayama, Kanagawa	35.266408	139.567093	Boulder	adhesion	9	0	○

49	2018/9/17	Tsuruoka, Yamagata	38.580805	139.557143	Rock	adhesion	1	0	○	
50	2018/9/24	Tobishima, Yamagata	39.183950	139.542082	Rock	adhesion	1	0	○	
51	2018/7/30	Sumoto, Hyogo	34.293238	134.947920	Rock	adhesion	8	0	○	
52	2018/10/12	Tsushima, Nagasaki	34.290861	129.261791	Boulder	adhesion	10	1	○	
53	2018/8/1	Tatsuno, Hyogo	34.771885	134.554413	Rock	adhesion	11	1	○	
54	2018/9/12	Shikamachi, Ishikawa	37.124282	136.727028	Rock	adhesion	6	0	×	
55	2018/11/21	Takahamacho, Fukui	35.534640	135.517240	Rock	adhesion	2	0	○	
56	2018/12/10	Shikamachi, Ishikawa	37.094815	136.726035	Rock	adhesion	1	0	○	
57	2018/12/22	Nanao, Ishikawa	37.128283	137.051020	Rock	adhesion	1	0	○	
58	2018/12/22	Nanao, Ishikawa	37.128283	137.051020	Boulder	adhesion	1	0	×	Hermit crabs that use the shell of *L. correensis* with *P. conchopheria*
59	2018/12/22	Nanao, Ishikawa	37.128283	137.051020	Boulder	adhesion	-	-	×	Hermit crabs that use the shell of *L. correensis* with *P. conchopheria*
60	2019/1/20	Tokitsu, Nagasaki	32.842296	129.851293	Rock	adhesion	3	0	○	
61	2019/3/9	Saikai, Nagasaki	33.056322	129.662356	Rock	adhesion	3	0	○	
62	2019/3/18	Nishio, Aichi	34.715525	137.047947	Sand	adhesion	1	0	○	
63	2019/4/19	Jeju, Jeju-teukbyeoljachido	33.548380	126.656135	Boulder, mud	adhesion	7	0	○	
64	2019/5/4	Sasebo, Nagasaki	33.218047	129.552842	Rock	adhesion	1	0	○	

65	2019/5/7	Tsuruga, Fukui	35.704700	136.079066	Rock	adhesion	14	14	×	
66	2019/5/12	Fukui, Fukui	36.102339	136.035040	Rock	adhesion	-	-	×	
67	2019/5/18	Hayama, Kanagawa	35.281940	139.568434	Boulder	adhesion	-	-	×	Hermit crabs that use the shell of *L. correensis* with *P. conchopheria*
68	2018/4/29	Mitoyo, Kagawa	34.191444	133.644222	Sand	No adhesion	0	2	○	
69	2018/8/2	Shirahama, Wakayama	33.691944	135.333611	Rock	No adhesion	0	1	○	
70	2018/8/3	Shirahama, Wakayama	33.487778	135.794722	Boulder	adhesion	1	0	○	
71	2018/9/1	Tahara, Aichi	34.656286	137.168237	Rock, Boulder	adhesion	2	0	○	
72	2019/9/15	Hamamatsu, Shizuoka	34.699827	137.605229	Sand	adhesion	2	0	○	
73	2019/9/19	Shimoda, Shizuoka	34.709889	138.984139	Rock	adhesion	4	0	○	
74	2019/10/29	Higashimatsushima, Miyagi	38.331050	141.146560	Sand	adhesion	2	0	○	
75	2019/10/12	Tateyama, Chiba	34.975824	139.775499	Sand, Rock	adhesion	-	-	×	
76	2019/4/22	Hayama, Kanagawa	35.273203	139.569414	Rock	adhesion	3	0	○	
77	2019/11/7	Numazu, Shizuoka	35.021016	138.896222	Rock	adhesion	4	0	○	
78	2019/7/15	Sumoto, Hyogo	34.292306	134.951389	Sand, Boulder	adhesion	6	0	○	
79	2020/3/14	Awaji, Hyogo	34.248456	134.720916	Rock	adhesion	2	0	○	
80	2019/7/1	Miura, Kanagawa	35.142951	139.664961	Rock	adhesion	8	0	○	
81	2019/7/1	Miura, Kanagawa	35.143082	139.664361	mud	adhesion	0	11	○	

82	2020/5/26	Futtsu, Chiba	35.167795	139.819445	Rock	adhesion	1	0	◯
83	2019/8/17	Miura, Kanagawa	35.153939	139.607181	Rock	adhesion	6	0	×
84	2019/6/19	Suzu, Ishikawa	37.485434	137.109061	Rock	adhesion	1	0	◯
85	2020/7/27	Matsumae, Hokkaido	41.554771	139.986097	Rock	adhesion	4	0	◯
86	2019/3/28	Numazu, Shizuoka	35.021490	138.897246	Rock	adhesion	2	1	◯
87	2019/10/6	Fukui, Fukui	36.114722	136.042778	Rock	adhesion	4	0	◯
88	2019/10/6	Fukui, Fukui	36.085098	136.029700	Rock	adhesion	3	0	◯
89	2018/10/17	Tateyama, Chiba	34.975885	139.774954	Rock	adhesion	1	0	×

Supplementary Table S2

Results of hierarchical Bayesian modeling using MCMC. Parameter values not shown in Table 1.

Parameter	Mean	S. D.	95% Bayesian confidence interval	
			Lower (2.5%)	Upper (97.5%)
ϕ	2.44	0.25	2.01	2.98
σ	1.54	0.45	0.83	2.55
σt	2.82	3.40	0.03	12.30
ssn_{spring}	-0.50	0.33	-1.14	0.15
ssn_{summer}	-0.26	0.32	-0.88	0.36
ssn_{autumn}	0.08	0.41	-0.68	0.92
ssn_{winter}	0.68	0.60	-0.53	1.82
$\beta_{4,1}$	0.72	0.69	-0.44	2.13
$\beta_{4,2}$	-0.39	0.48	-1.34	0.52
$\beta_{4,3}$	-0.03	0.50	-1.07	0.93
$\beta_{4,4}$	-0.08	0.23	-0.50	0.34
$\beta_{4,1} - \beta_{4,2}$	1.11	0.88	-0.34	3.04
$\beta_{4,1} - \beta_{4,3}$	0.75	0.87	-0.73	2.63
$\beta_{4,1} - \beta_{4,4}$	0.80	0.71	-0.40	2.27
$\beta_{4,2} - \beta_{4,3}$	-0.36	0.64	-1.67	0.82
$\beta_{4,2} - \beta_{4,4}$	-0.31	0.53	-1.42	0.63
$\beta_{4,3} - \beta_{4,4}$	0.05	0.53	-1.19	1.06

Supplementary Figure S1

Lunella correensis preyed on by the molluscan predators, *Hemifusus ternatanus* in the rearing environment. These images were reported in this project by research participant, a middle school student.

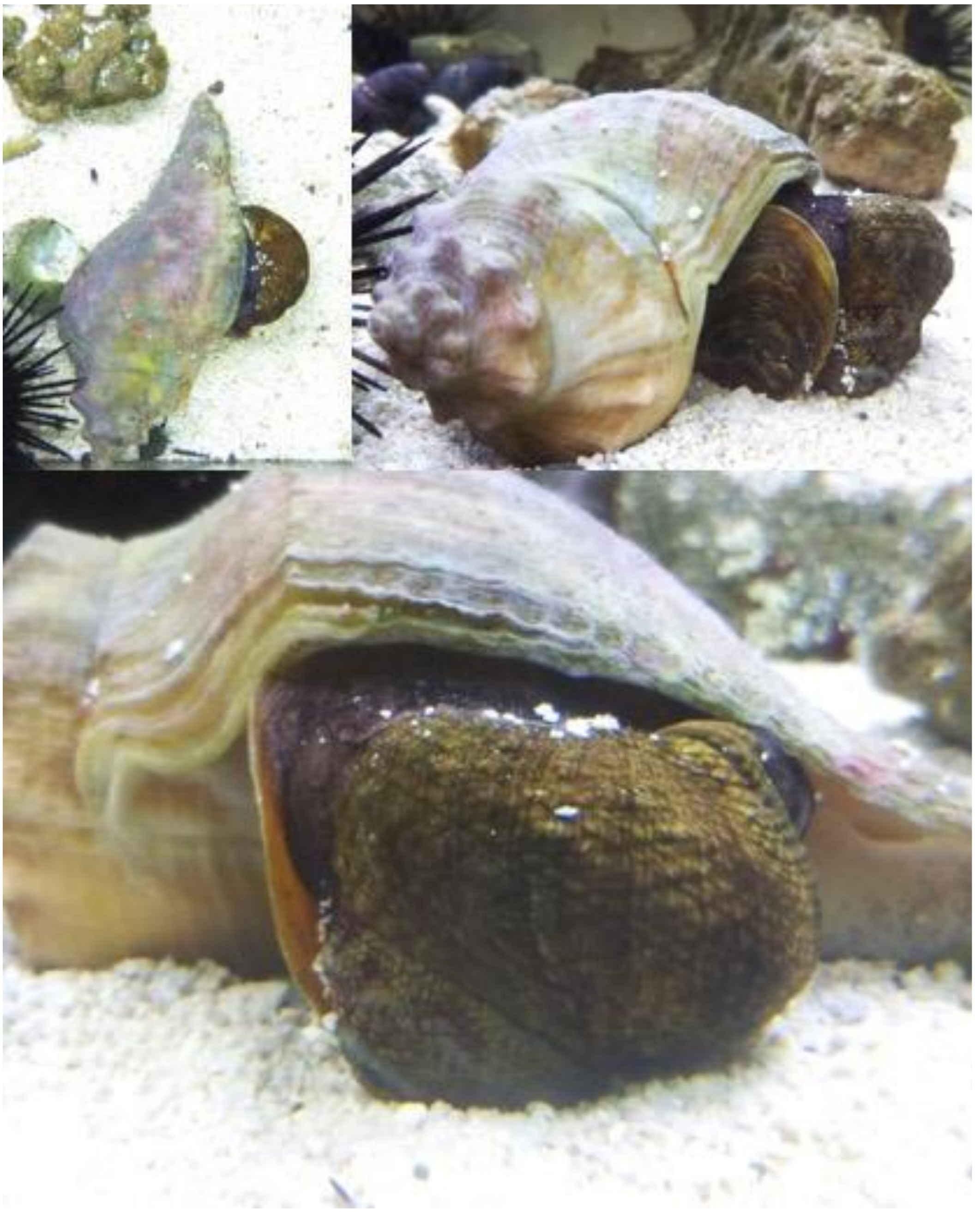

Supplement: Supplementary file 1 — Supplementary Information [file 41598_2020_74946_MOESM1_ESM.pdf]
